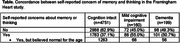# Self‐awareness of cognitive impairment in a community‐based cohort

**DOI:** 10.1002/alz.089515

**Published:** 2025-01-09

**Authors:** A. M. Barrett, Ting Fang Alvin Ang, Yulin Liu, Sherral A. Devine, Jesse Mez, Rhoda Au, Honghuang Lin

**Affiliations:** ^1^ UMass Chan Medical School, Worcester, MA USA; ^2^ Boston University Chobanian & Avedisian School of Medicine, Boston, MA USA; ^3^ Framingham Heart Study, Boston University Chobanian & Avedisian School of Medicine, Boston, MA USA; ^4^ Boston University Chronic Traumatic Encephalopathy Center, Boston University Chobanian & Avedisian School of Medicine, Boston, MA USA

## Abstract

**Background:**

The deficit of unawareness of cognitive impairment (cognitive anosognosia) is known to be associated with adverse health outcomes, caregiver burden, and worse cognitive outcomes. A better understanding of cognitive self‐awareness and the ability to self‐judge cognitive performance among the general population would enable a rational design of cognitive screening and improve how subjective cognitive decline and self‐reported errors at tasks like medication administration are interpreted.

**Method:**

Participants were enrolled in the Framingham Heart Study, which is a community‐based cohort with three generations of participants. Through ancillary studies, FHS participants underwent neuropsychological assessments every 4 to 5 years. For those with possible cognitive impairment, study examiners identified, and a panel later adjudicated, a clinical diagnosis (intact, mild cognitive impairment [MCI], or dementia, and their subtypes). Before the neuropsychological testing, participants were asked, “Do you have any concerns about your memory or thinking”. If “yes,” they were further asked: “Do you think this is normal for your age?”

**Result:**

Our study included 5,110 participants of FHS with self‐reported assessment of cognition (mean age: 67±15 years; 55.1% women, 66.2% with some college degree or above). As shown in **Table 1**, among 359 (7.0% of the participants) diagnosed with MCI or dementia, 47.4% (170) had a self‐report consistent with unawareness of cognitive impairment (72 or 45.0% of MCI, 98 or 49.2% of dementia). These individuals had NO self‐reported concerns at all about memory and thinking. Even more concerning, a larger group of people (p = 0.025) with MCI or dementia who DID self‐report concerns about memory/thinking (88 with MCI, or 55.0%, 101 with dementia, 50.7%), stated that they thought the problem was normal for their age (124 participants, 65.6%).

**Conclusion:**

Most of the Framingham Heart Study participants clinically diagnosed with pathologic cognitive impairment self‐reported that they either had no memory or thinking problems, or, more often, self‐reported memory and thinking problems that they felt were normal for their age. This is consistent with cognitive anosognosia, which can represent a range of unawareness. Since subjective cognitive decline is used to identify MCI, further research could develop complementary MCI diagnosis markers.